# By inhibiting snail signaling and miR-23a-3p, osthole suppresses the EMT-mediated metastatic ability in prostate cancer

**DOI:** 10.18632/oncotarget.4229

**Published:** 2015-05-22

**Authors:** Yu-Ching Wen, Wei-Jiunn Lee, Peng Tan, Shun-Fa Yang, Michael Hsiao, Liang-Ming Lee, Ming-Hsien Chien

**Affiliations:** ^1^ Graduate Institute of Clinical Medicine, College of Medicine, Taipei Medical University, Taipei, Taiwan; ^2^ Department of Urology, Wan Fang Hospital, Taipei Medical University, Taipei, Taiwan; ^3^ Institute of Medicine, Chung Shan Medical University, Taichung, Taiwan; ^4^ Department of Medical Research, Chung Shan Medical University Hospital, Taichung, Taiwan; ^5^ The Genomics Research Center, Academia Sinica; Taipei, Taiwan; ^6^ Department of Education and Research, Wan Fang Hospital, Taipei Medical University, Taipei, Taiwan

**Keywords:** prostate cancer, miR-23a-3p, snail, EMT, osthole

## Abstract

Here we showed that Osthole, 7-methoxy-8-(3-methyl-2-butenyl) coumarin, a bioactive coumarin derivative extracted from medicinal plants, inhibited migration, invasion, epithelial to mesenchymal transition (EMT) in androgen-independent prostate cancer (AIPC) cells *in vitro* and metastasis of AIPC *in vivo*. In patients, high Snail levels were correlated with a higher histological Gleason sum and poor survival rates. Osthole inhibited the TGF-β/Akt/MAPK pathways, reduced Snail-DNA-binding activity and induced E-cadherin. We found that osthole decreased miR-23a-3p. Ectopic miR-23a-3p suppressed E-cadherin 3′ untranslated region reporter activity and E-cadherin expression, and relieved the motility suppression caused by osthole treatment.

## INTRODUCTION

Prostate cancer is the second leading cause of cancer-related deaths among males in the USA [1, 2]. Currently, androgen-depletion therapy (ADT) is the predominant treatment, which is effective for androgen-dependent prostate cancer (ADPC). However, prostate cancer usually progresses from an androgen-dependent to an androgen-independent stage, making anti-androgen therapy ineffective and leading to an increase in the metastatic potential and an incurable malignancy, the so called androgen-independent prostate cancer (AIPC) or castration-resistant prostate cancer (CRPC) [3]. Chemotherapy has not shown satisfactory results for prostate cancer. For example, patients with metastatic AIPC treated with docetaxel can only maintain progression-free survival for less than 6 months [4]. Some natural compounds, such as psoralidin and FTY720, or chemical compounds, such as non-steroidal anti-inflammatory drugs (NSAIDs) and tasquinimod, possess chemopreventive effects [5–8].

The majority of deaths associated with prostate cancer are attributed to a failure to cure metastatic disease [9]. The epithelial-mesenchymal transition (EMT) is one of events in cancer metastasis and often occurs at the invasive front of tumors [10]. During EMT, cells lose their epithelial traits and acquire mesenchymal characteristics, such as downregulating E-cadherin (E-cad) and β-catenin, and upregulating N-cadherin (N-cad) and fibronectin, resulting in a weakened adhesion ability and enhanced motility [11]. EMT confers chemoresistance in several cancer types [12]. Growth factors, such as transforming growth factor (TGF)-β and epidermal growth factor (EGF), induce EMT by modulating these epithelial (E-cad) and mesenchymal markers (N-cad) [13]. Several transcription factors, including Snail, a zinc finger transcription factor, control EMT [14]. Snail binds to the promoter of the *E-cad* gene and represses its transcription, which is one of the hallmark events in EMT and metastasis [15–17]. Ectopic expression of Snail triggers the EMT, enhances cancer cell motility, and confers resistance to senescence of cancer cells [18], which provides a selective advantage for tumors that become malignant.

Micro (mi)RNAs are single-stranded, noncoding endogenous RNAs, of approximately 18∼24 nucleotides long, which post-transcriptionally modulate gene expression by either inhibiting translation or inducing messenger (m)RNA degradation. miRNAs function as oncogenes or tumor-suppressor genes through respectively binding to 3′ untranslated regions (UTRs) of target tumor suppressor genes or oncogenes [19, 20]. In particular, miRNAs are involved in EMT [21, 22]. Array-based miRNA profiling of human cancer cells identified an association between miRNA deregulation and cancer metastasis [23–25].

Osthole, 7-methoxy-8-(3-methyl-2-butenyl) coumarin, a simple bioactive coumarin derivative extracted from medicinal plants such as *Cnidium monnieri* (L.) Cusson, inhibits growth and metastasis in various cancer types [26–28]. Coumarin was also used in a clinical trial to prevent disease recurrence in melanoma patients [29]. However, compared to other tumor types, data regarding the antitumor effects of osthole on AIPC are largely unknown. Thus, in the present study, we investigated the effects of osthole on the motility of AIPC cells and elucidated possible underlying molecular mechanisms. We showed that osthole suppresses the EMT-mediated metastatic potential of human AIPC through transcriptional and epigenetic regulation of the EMT-related molecule, E-cad, by respectively inhibiting the TGF-β/Akt/MAPK/Snail signal cascade and downregulating miR-23a-3p.

## RESULTS

### Suppression of migration and invasion of human AIPC cells by osthole treatment

**Figure 1:**
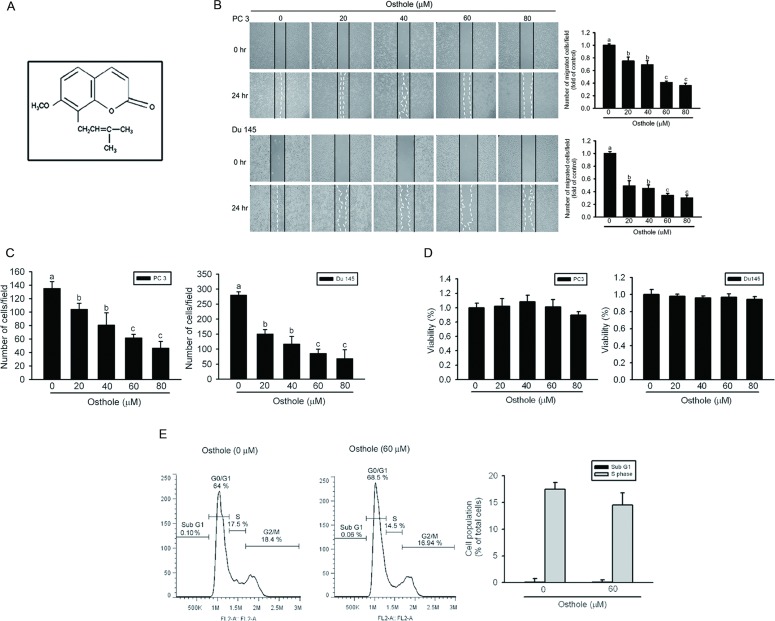
Effects of osthole on cell migration, invasion, and viability in human androgen-independent prostate cancer (AIPC) cells **A.** The chemical structure of osthole. **B.** The effect of osthole on wound closure in AIPC cells. Two AIPC cell lines, PC3 and DU145, were wounded and then treated with the vehicle or osthole (20~80 μM) for 24 h, and phase-contrast pictures of the wounds were taken (left panel). Cells migrating into the wound area were counted using the dashed line as time zero. A quantitative assessment of the mean number of cells in the denuded zone is presented as the mean ± SE (*n* = 3) (right panel). **C.** AIPC cells were treated with the indicated concentrations of osthole for the Matrigel invasion assays. Values are presented as the mean ± SE (*n* = 3). Data from the wound-closure and invasion assays were analyzed using a one-way ANOVA with Tukey's post-hoc tests at 95% confidence intervals; different letters represent different levels of significance. **D.** The effect of osthole on the viability of AIPC cells. DU145 and PC3 cells were treated with the vehicle or osthole (20~80 μM) in serum-containing medium for 24 h. Cell viability was determined with an MTS assay. **E.** DU145 cells were released from quiescence by incubation in culture medium supplemented with 10% FBS and the vehicle or osthole (60 μM) for 24 h. Cells were harvested, and cell-cycle distributions in the sub-G_1_ and S-phases were determined by a FACS analysis.

The chemical structure of osthole is shown in Figure 1A. To study the effect of osthole on prostate cancer cell migration and invasion abilities, we respectively performed wound-closure and Matrigel invasion assays on two metastatic AIPC cell lines, DU145 and PC3. After treating DU145 or PC3 cells with various concentrations of osthole for 24 h, results showed that osthole concentration-dependently suppressed the wound-closure and invasive abilities at concentrations of 20~80 μM (Figure 1B, 1C). To exclude the possibility that decreased numbers of migrating and invading cells were a consequence of reduced proliferation, we performed viability assays using DU145 and PC3 cells treated with the same osthole concentration that was used in the wound-closure and invasion assays. Results from the MTS assay showed that even at the highest concentration of 80 μM, osthole only partially altered or did not alter the viability of these AIPC cell lines with 24-h treatment, compared to that of the controls (Figure 1D). We further investigated the effects of osthole on cell cycle regulation. Results showed that treatment of DU145 cells with osthole (60 μM) for 24 h did not increase the incidence of apoptosis. The lack of significant changes in the sub G_1_ population between control and osthole-treated DU145 cells provides evidence for this (Figure 1E). The DU145 cell proliferation rate was also not affected by osthole, because the number of cells in the S-phase did not significantly change after 24 h of osthole treatment (Figure 1E). According to these data, osthole significantly inhibited cancer cell invasion at a non- or low-cytotoxic concentration, indicating that osthole is an effective inhibitor of the motility of AIPC cells.

### Significant antimetastatic and antiproliferative effects of osthole in a PC3 orthotopic graft model

**Figure 2:**
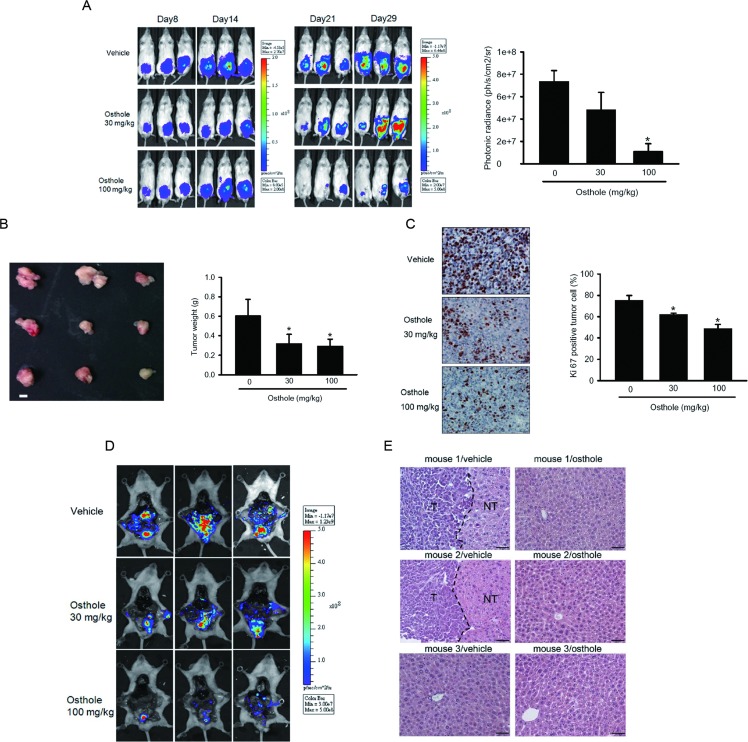
Significant antimetastatic and antiproliferative effects of osthole in a PC3M orthotopic graft model Luciferase-tagged PC3M cells were injected into the prostate of SCID mice (three mice in each group). Eight days after tumor cell injection, mice were treated with osthole (40 or 100 mg/kg, IP) or the vehicle every other day. The tumor size was monitored by bioluminescence imaging at the indicated time intervals. Twenty-one days after osthole treatment, animals were sacrificed, and tumor specimens were collected. **A.** Left panel, Xenogen IVIS spectrum bioluminescence imaging of orthotopic prostate tumor growth. Right panel, Quantitative analysis of Xenogen imaging signal intensity (photons/sec/cm^2^/steradian) at the end of the study with the mean signal for each group indicated. * *p* < 0.05, compared to the vehicle groups. **B.** Left panel, Gross appearance of orthotopic tumors after treatment with the vehicle or osthole for 21 days. Scale bar = 1 cm. Right panel, The average tumor weight of each group is shown. * *p* < 0.05, compared to the vehicle groups. **C.** A proliferation index was determined based on Ki67 immunostaining, and Ki67-positive cells were counted at ×200 magnification per PC3M tumor section (left panel). The mean value of the Ki67 expression percentage in each group is shown in the right panel. * *p* < 0.05, compared to the vehicle groups. **D.** Cancer metastasis including proximal invasion and distal metastasis was imaged with bioluminescence at the end of the study. **E.** Histological analyses of liver metastasis from the vehicle or osthole-treated group. T, tumor part; NT, non-tumor part.

Although the antitumor effect of osthole was demonstrated in various cancer types *in vitro* [26, 28], studies on its antitumor effects *in vivo* are rare. Here, luciferase-expressing PC3M-Luc cells were established and injected into the capsule of the anterior prostate of SCID mice and allowed to become established for 8 days before initiating treatment. PC3M-Luc orthotopic graft mice were treated with different dosages of osthole or the vehicle control every other day by IP administration, and tumor growth and metastasis were monitored by bioluminescence imaging. Figure 2A shows the inhibitory potency of osthole on tumor growth after 21 days of treatment by photon emission detection *in vivo*. In osthole-treated mice receiving 100 mg/kg, the mean tumor volume on day 21 was significantly inhibited compared to vehicle-treated PC3M tumors (Figure 2A). Mice were sacrificed at the end of the experiment (29 days after cell injection), and the mean tumor weight was lower in 30- and 100-mg/kg osthole-treated mice compared to vehicle-treated mice (Figure 2B). To examine whether osthole exerts antitumor activity *in vivo* via inhibiting proliferation, proliferating cells were indicated by immunocytochemical staining of the proliferation marker, Ki67. After treatment with osthole, numbers of Ki67-positive cells were reduced compared to control mice (Figure 2C). In addition to tumor growth inhibition, formation of spontaneous metastasis was dramatically prevented dose-dependently by osthole according to evidence from *ex vivo* photon imaging which indicated that the organs around the prostate showed a lower intensity in 30- and 100-mg/kg osthole-treated mice compared to vehicle-treated mice (Figure 2D). In addition, H&E staining revealed that the incidence of distal liver tumor metastasis in vehicle-treated mice was higher than that in 100-mg/kg osthole-treated mice (Figure 2E). These observations suggest that osthole was an effective agent that inhibited the growth and metastasis of transplanted prostate gland tumors *in vivo*.

### Osthole suppresses Snail-mediated EMT progression in AIPC

**Figure 3:**
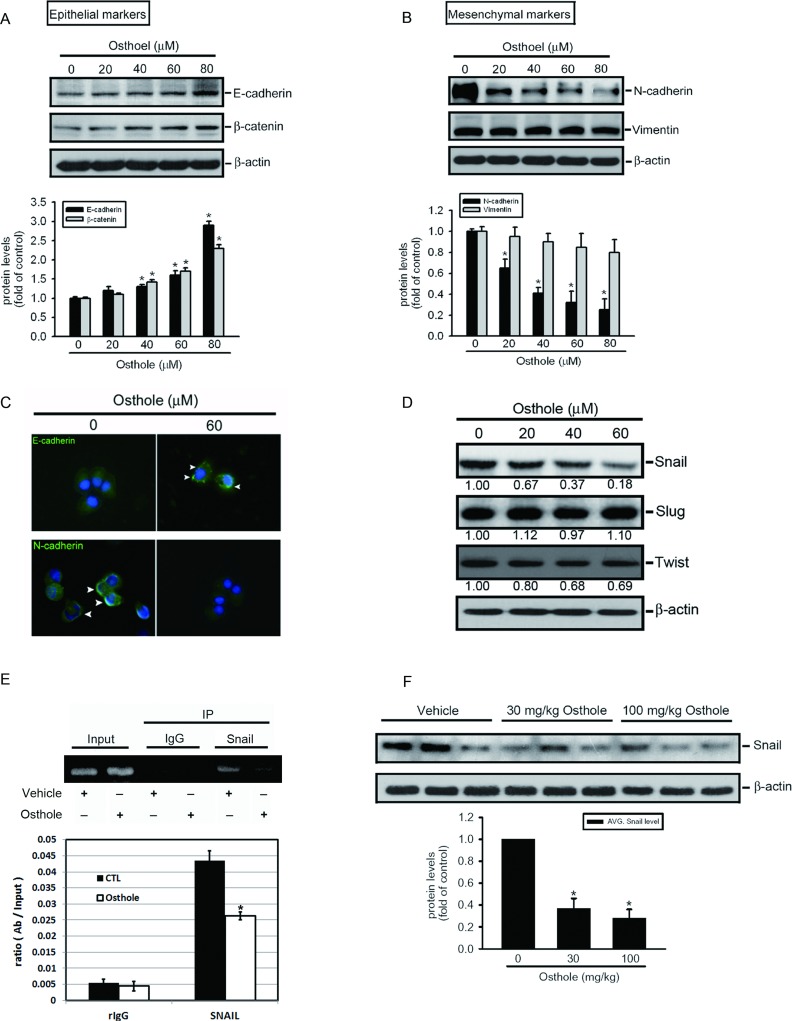
Osthole attenuates the snail-mediated epithelial-mesenchymal transition (EMT) in Du145 cells and in the PC3M orthotopic graft model **A.**, **B.** Epithelial markers, E-cadherin and β-catenin, **A.** and mesenchymal markers, N-cadherin and vimentin, **B.** were expressed in osthole-treated DU145 cells. Lysates were collected from cells cultured with or without different concentrations of osthole (0~80 μM) for 24 h and subjected to a Western blot analysis. Quantitative results of EMT-related protein levels, which were adjusted to the β-actin protein level. Values are presented as the mean ± SE of three independent experiments. * *p* < 0.05, compared to the vehicle groups. **C.** Immunofluorescence staining depicting losses of E-cadherin levels and increases of N-cadherin levels in the plasma membrane of DU145 cells after treatment with 60 μM osthole for 24 h. **D.** Expression levels of EMT-related transcriptional factors in osthole-treated AIPC cells were measured from lysates collected from DU145 cells cultured with different concentrations of osthole (0~80 μM), with changes in quantities shown with the indicated bands. **E.** ChIP analysis of the association of the transcription factor, Snail, with the E-cad promoter region in DU145 cells. Upper panel, ChIP assays were conducted on DU145 cells using a Snail antibody to screen the Snail-bound E-cad promoter region for PCR amplification. Lower panel, The effect of osthole on the DNA-binding activity of Snail was confirmed by a ChIP-qPCR assay. IgG was used as a negative control. Values are presented as the mean ± SE of three independent experiments. * *p* < 0.05, compared to the vehicle groups. **F.** Upper panel, PC3M orthotopic tumors isolated for protein extraction at 21 days after the vehicle or osthole treatment and subjected to a Western blot analysis. Lower panel, Quantitative results of Snail protein levels, which were adjusted to the β-actin protein level. Values are presented as the mean ± SE (*n*=3). * *p* < 0.05, compared to the vehicle groups.

Recently, newly emerging evidence indicates that the EMT and degradation of the extracellular matrix (ECM) play crucial roles during the development and metastasis of AIPC [30, 31]. Effects of osthole on the EMT and ECM degradation of AIPC cells were examined by treating DU145 cells with various concentration of osthole for 24 h and observing EMT- and ECM degradation-related proteins. Western blotting (Figure 3A, 3B) and immunofluorescent staining (Figure 3C) results showed that treatment with osthole increased expressions of the epithelial markers, E-cad and β-catenin, and decreased expression of the mesenchymal marker, N-cad, in DU145 cells, but had no effect on the activities of ECM degradation-related proteins, MMP-2 and MMP-9 (Supplementary Figure S1A). The undetectable effect of osthole on MMP-2/9 activation and similar effect of osthole on EMT-related markers expression were also observed in PC3 cells (Supplementary Figure S1B and Figure S2). The molecular signals involved in osthole-mediated suppression of the EMT were elucidated by investigating expressions of several EMT-related transcriptional factors (Snail, Slug, and Twist) which are recognized as transcriptional repressors of E-cad [32]. Treatment with osthole significantly decreased the expression of Snail in DU145 cells, but had less or undetectable effects on the other transcriptional factors, Twist and Slug, respectively (Figure 3D). Moreover, a ChIP assay was performed to investigate the effect of osthole on the binding of Snail to the E-cad promoter. As illustrated in the upper panel of Figure 3E, binding of Snail to the E-cad promoter decreased in DU145 cells after osthole treatment. This result was further confirmed by a quantitative real-time PCR assay; osthole indeed significantly suppressed binding of Snail to the E-cad promoter (Figure 3E, lower panel). We further detected Snail protein levels of PC3M xenografts harvested from vehicle- or osthole-treated mice and found that levels of Snail had also decreased in tumors isolated from osthole-treated mice (Figure 3F). These data suggest that downregulating Snail is important for the osthole-induced suppression of the EMT in AIPC cells and an AIPC xenograft model.

### The TGF-β/Akt/MAPK signaling cascade is involved in osthole's suppression of Snail expression

**Figure 4:**
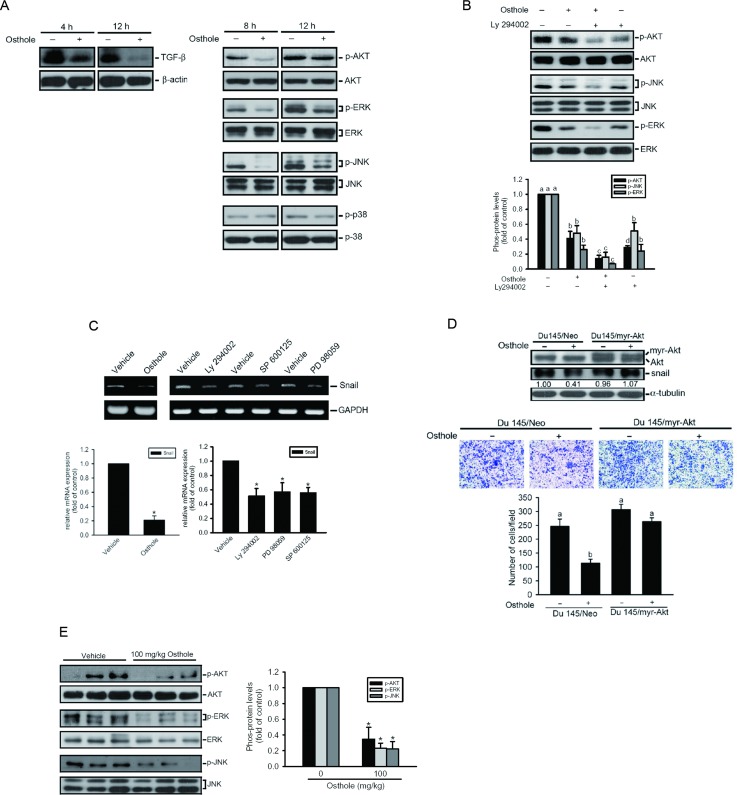
The transforming growth factor (TGF)-β/AKT/mitogen-activated protein kinase (MAPK) pathways are involved in osthole-mediated suppression of Snail expression, and cell motility **A.** Expression level of TGF-β and phosphorylation (p) levels of Akt, extracellular signal-regulated kinase (ERK)1/2, p38, and c-Jun N-terminal kinase (JNK)1/2 were assessed by a Western blot analysis after treatment of DU145 cells with osthole (60 μM) for the indicated time points. **B.** DU145 cells were pretreated with or without Ly294002 (20 μM) for 1 h followed by osthole (60 μM) treatment for an additional 8 h. Phosphorylation levels of Akt, ERK1/2, and JNK1/2 were assessed by a Western blot analysis. Quantitative results of p-Akt, p-ERK, and p-JNK1/2 protein levels, which were respectively adjusted to their total protein levels. Values are presented as the mean ± SE of three independent experiments. Different letters represent different levels of significance. **C.** Expressions of p-Akt, p-ERK, and p-JNK1/2 protein levels in PC3M orthotopic tumors which were treated with the vehicle or osthole for 21 days. Quantitative results of p-Akt, p-ERK, and p-JNK1/2 protein levels, which were respectively adjusted to their total protein levels. Values are presented as the mean ± SE (*n*=3). * *p* < 0.05, compared to the vehicle groups. **D.** DU145 cells were treated with osthole (60 μM), Ly294002 (20 μM), SP600125 (20 μM), or PD98059 (25 μM) for 6 h and then subjected to an RT-PCR to analyze Snail mRNA expression. Values are presented as the mean ± SE of three independent experiments. * *p* < 0.05, compared to the vehicle groups. **E.** Upper panel, Western blot analysis of Akt and Snail expressions in DU145 cells expressing myr-Akt. Changes in Snail values are shown by the indicated bands. Lower panel, Invasive ability of DU145/myr-Akt or DU145/Neo which was treated with the vehicle or osthole. Multiples of differences are presented as the mean ± SE of three independent experiments. Data were analyzed using a one-way ANOVA with Tukey's post-hoc tests at 95% confidence intervals; different letters represent different levels of significance.

TGF-β is a potent EMT inducer via the PI3K/Akt pathway during tumor progression [33]. Activation of PI3K/Akt was reported to play an important role in prostate tumorigenesis and its progression to castration resistance, indicating that an Akt-elated pathway may play an important role in AIPC. Higher levels of p-MAPK were observed in malignant and AIPC prostate tissues than in non-malignant specimens [30]. We therefore examined whether TGF-β production and the Akt and MAPK pathways were involved in osthole-mediated suppression of Snail and cell invasion in AIPC cells. Our results showed that treatment of Du145 cells with osthole for different time points (4~12 h) suppressed expression of TGF-β at an earlier phase (4 h after treatment) and activation of Akt, JNK1/2, and ERK1/2 at a later phase (8 h after treatment) (Figure 4A). Moreover, pretreatment of Du145 cells with the PI3K/Akt inhibitor, Ly294002, enhanced the inhibitory effect of osthole against JNK1/2 and ERK1/2 activities, indicating that the JNK and ERK pathways are downstream signals of Akt (Figure 4B). Next, we further investigated relationships among osthole-mediated inhibitory effects on Akt/MAPK activation, Snail expression, and the cell-invasive ability. Treatment of Du145 cells with a MEK/ERK inhibitor (PD 98059), a JNK inhibitor (SP 600125), or Ly294002 significantly inhibited Snail expression (Figure 4C). The constitutively activated Akt, myr-Akt, was ectopically expressed in Du145 cells (Figure 4D, upper panel) to further investigate whether Akt plays an important role in osthole-mediated repression of Snail and cell motility in AIPC. As shown in the lower panel of Figure 4D, overexpressing activated Akt in Du145 cells partially increased the ability of cells to invade. Overexpressing activated Akt significantly rescued the osthole-mediated suppression of Snail expression (Figure 4D, upper panel) and the cell-invasive ability (Figure 4D, lower panel). Moreover, suppressive effects of osthole on activation of Akt, JNK, and ERK were also observed in osthole-treated PC3 grafts (Figure 4E). Taken together, these results suggest that osthole mediated downregulation of E-cad's transcriptional repressor, Snail, and cell-invasive ability by suppressing the TGF-β/Akt/MAPK signaling cascade in AIPC cells.

### Biological significance of Snail in prostate cancer progression

**Figure 5:**
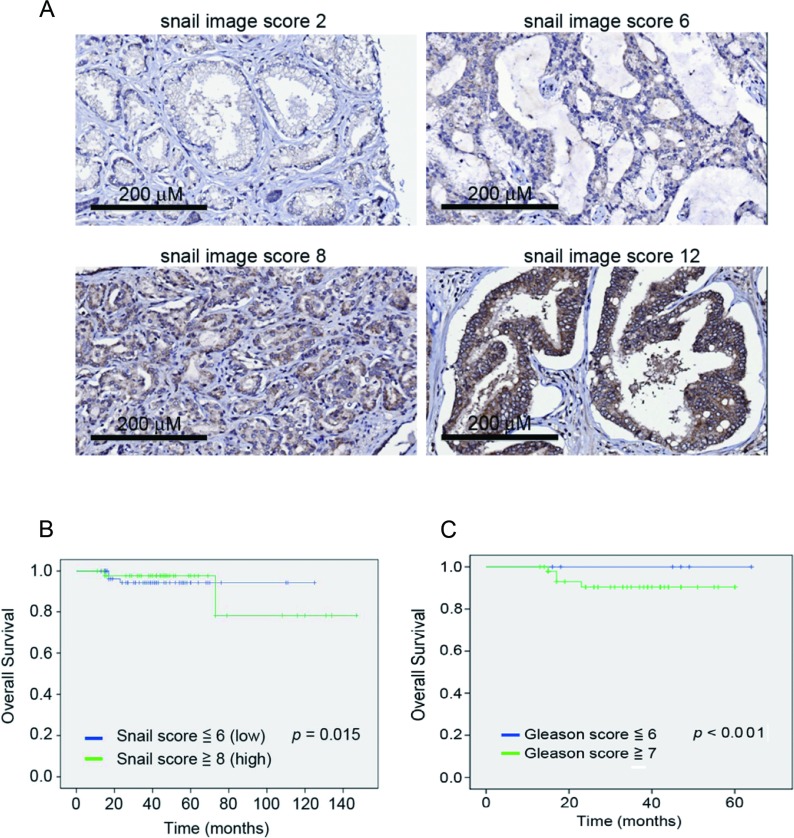
Clinical significance of Snail in prostate cancer progression **A.** Tissue microarrays of primary prostate cancers (110) were immunohistochemically analyzed for Snail. Representative data respectively show patients with a weak Snail expression level (intensity score 1 × extent score 2 = Snail image score 2), a moderate Snail expression level (intensity score 2 × extent score 3 = Snail image score 6), a marked snail expression level (intensity score 2 × extent score 4 = Snail image score 8), and a heavily dominant snail expression level (intensity score 3 × extent score 4 = Snail image score 12) in the nucleus (×200). **B.**, **C.** Kaplan-Meier survival curve showing relationships of the Snail image score **A.** and Gleason score sum **B.** in primary tumors with overall survival in 110 patients with prostate cancer. The survival rate of patients with a higher snail image score (≥ 8) or Gleason score sum (≥ 7) was significantly lower than that of patients with a lower snail image score (≤ 6) or Gleason score sum (≤ 6) (*p* < 0.05, log-rank test).

In addition to defining the role of Snail in osthole-mediated suppression of cell motility *in vitro* and *in vivo*, we further determined the prognostic significance of Snail in 110 human prostate cancer specimens that had clinical follow-up records. The clinicopathological features of these 110 patients are shown in Table 1. Representative examples with different Snail scores are shown in Figure 5A. Relationships between Snail expression levels and clinicopathological characteristics of prostate cancer are summarized in Table 2. Among these specimens, high Snail expression levels (with a score of ≥ 8) were strongly correlated with a higher histological Gleason score and a higher preoperative PSA value compared to tumors with low-Snail expression levels (with a score of ≤ 6). With the Kaplan-Meier log rank test, we observed that patients with high Snail expression and a high Gleason score sum (of ≥ 7) had reduced overall survival times (Figure 5B, 5C). Our data indicated that higher levels of Snail predicted a poor prognosis in prostate cancer.

**Table 1:** Characteristics of 110 clinical T2 prostate cancer patients who underwent a radical prostatectomy (RP)

Characteristic	Total (%)
Total number of patients	110
Mean preoperative PSA level (ng/ml)	22.9 (0.5-161)
Gleason score sum	
≧7	82(74.5 %)
≦6	28(25.5 %)
Pathological stage	
II	58 (52.7 %)
III	51 (46.3 %)
IV	1 (1.0 %)
Snail image score	
≧8 (high)	46(41.8 %)
≦6 (low)	64(48.2 %)
Survival (Pt. No.)	
Snail score high	43
Snail score low	61
Mean fellow-up time (months)	46.5(11-147)

**Table 2:** Prostate cancer patient characteristics according to snail expression

Characteristic	No. of patients (%)		*p* value
	Snail score ≥ 8	Snail score ≤ 6	
**Total number of patients**	46 (66)	64 (34)	
**Pathological stage**			
II	28(60.9)	30(46.9)	0.56^a^
III+IV	18(39.1)	34(53.1)	
**Gleason score**			
≥7	39(84.8)	42(65.6)	0.001^a^
≤6	7(15.2)	22(34.4)	
**PSA, mean (ng/ml)**	30.61	5.79	0.02^b^

**Abbreviations:** PSA: prostate specific antigen

a Chi-squared test

b T-test pairs

**Prostate cancer patient characteristics according to snail expression**

### Downregulation of miR-23a-3p is involved in osthole-mediated upregulation of E-cad and suppression of cell motility

**Figure 6:**
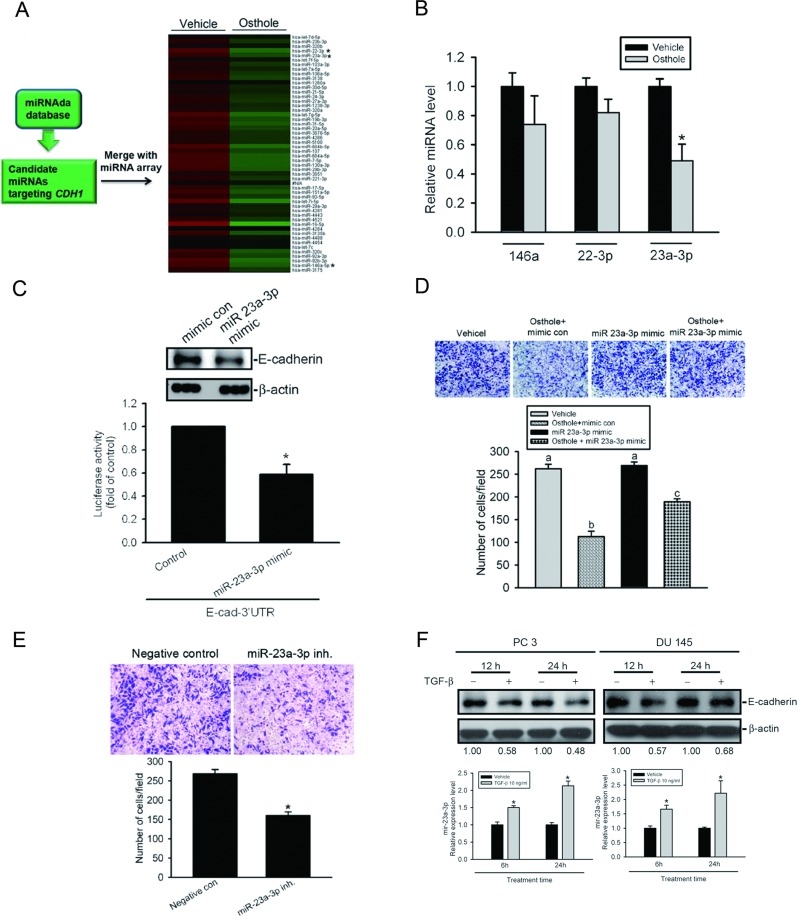
Downregulation of miR-23a-3p is involved in osthole-mediated upregulation of E-cadherin expression and inhibition of cell motility **A.** A schematic representation of the procedure for miRNA selection. Differential expressions of miRNAs in osthole-treated cells versus vehicle-treated cells were analyzed with a OneArray^®^ miRNA profiling chip. **B.** Treatment of Du145 cells with osthole for 6 h. miR-146a, miR-22-3p, and miR-23a-3p expressions were detected by a quantitative PCR. **C.** Upper panel, Du145 cells were transfected with an miR-23a-3p mimic or mimic control for 24 h followed by osthole (60 μM) treatment for an additional 24 h. E-cadherin expression levels were determined by a Western blot analysis. Quantitative E-cadherin protein levels were adjusted to the β-actin protein level. Lower panel, Relative luciferase activities of DU145 cells co-transfected with an E-cadherin luciferase 3′UTR reporter vector and miR-23a-3p mimic or mimic control for 24 h. Values are presented as the mean ± SE of three independent experiments. * *p* < 0.05, compared to the control groups. **D.** DU145 cells were transfected with an miR-23a-3p mimic or mimic control for 24 h followed by osthole (60 μM) treatment for an additional 24 h. The cell-invasion ability was determined by a Matrigel invasion assay. Values are presented as the mean ± SE of three independent experiments. Data were analyzed using a one-way ANOVA with Tukey's post-hoc tests at 95% confidence intervals; different letters represent different levels of significance. **E.** DU145 cells were transfected with either an miR-23a-3p inhibitor or a negative control. The cell-invasion ability was determined by a Matrigel invasion assay. Values are presented as the mean ± SE of three independent experiments. * *p* < 0.05, compared to the control groups. **F.** PC3 or DU145 cells were treated with TGF-β for 6, 12, or 24 h. Expression levels of E-cadherin and miR-23a-3p were determined by Western blotting (upper panel) and a quantitative PCR (lower panel), respectively. Quantitative E-cadherin protein levels were adjusted to the β-actin protein level.

miRNA was reported to be an important regulator of cancer progression and metastasis [34]. In addition to determining the Snail-mediated transcriptional regulation of *E-cad* by osthole, we next investigated whether miRNA participates in osthole-mediated upregulation of E-cad and inhibition of cell motility. To identify which miRNAs are regulated by osthole, a high-throughput and specific miRNA microarray (human miRNA OneArray^®^ miRNA profiling chip) using Du145 cells after osthole treatment was conducted by the Phalanx Biotech Group (Hsinchu, Taiwan). Expressions of miRNAs downregulated by osthole (60 μM) treatment were shown in a heat map, and the top 10 downregulated miRNAs were further analyzed by the miRNA target database, miRanda, to determine which miRNA might target E-cad (Figure 6A). We found that miR-146a, -22-3p, and -23a-3p were miRNAs downregulated in response to osthole treatment and might target E-cad. To further confirm the expressions of these three miRNAs, we performed a TaqMan quantitative real-time PCR analysis and observed that miR-23a-3p expression was most downregulated by osthole treatment (Figure 6B). Furthermore, we try to ectopically express miR-23a-3p in Du145 cells and found that the endogenous E-cad level was downregulated after overexpression of miR-23a-3p in Du145 cells (Figure 6C, upper panel). Next, to examine whether miR-23a-3p can directly target the 3′UTR of E-cad, we constructed a luciferase reporter vector harboring the 3′UTR of E-cad and transfected this vector combined with the miR-23a-3p mimic or mimic control into Du145 cells. Results showed that miR-23a-3p, but not the mimic control, decreased luciferase activity (Figure 6C, lower panel). These results suggested that miR-23a-3p can be suppressed by osthole in Du145 cells and directly represses E-cad expression through binding to the 3′UTR of the human *E-cad* gene. To further verify the direct effect of miR-23a-3p on cell motility, we ectopically expressed miR-23a-3p in DU145 cells, and found that the overexpression of miR-23a-3p significantly reversed osthole-mediated inhibition of cell invasion (Figure 6D). In contrast, when we transiently transfected the miR-23a-3p inhibitor into DU145 cells, the invasive ability was significantly downregulated compared to control cells (Figure 6E). Recent studies indicated that miR-23a-3p is directly induced by TGF-β in hepatocellular carcinoma (HCC) and lung cancer cells [21,35]. We treated AIPC cells (PC3 and Du145) cells with 10 ng/ml TGF-β for different time points (6~24 h) and found that E-cad downregulation was accompanied by the upregulation of miR-23a-3p (Figure 6F). Moreover, pretreatment of Du145 cells with osthole can significantly suppress TGF-β-induced upregulation of mir-23a-3p (Supplementary Figure S3). Taken together, our results indicate that osthole suppresses AIPC cell motility might be through downregulating TGF-β-mediated miR-23a-3p expression.

## DISCUSSION

Cadherin switching usually refers to a switch from the expression of E-cadherin (E-cad) to that of N-cadherin (N-cad). N-cad promotes motility when expressed by epithelial cells, and cells that express more E-cad are less motile [11]. Genetic or epigenetic alterations in the *E-cad* gene or alterations in its protein expression increases invasiveness of tumor cells and metastasis [36]. In the present study, osthole upregulated E-cad and downregulated N-cad in DU145 AIPC cells, and as a consequence, it may prevent TGF-β-induced migration and invasion by prostate cancer cells. To further understand the molecular signals behind osthole-induced E-cad expression, we studied the effect of osthole on the transcriptional repressors Snail, Slug, and Twist, which are implicated in controlling the EMT. Our study showed that the protein level of Snail exhibited a rapid decrease after osthole treatment in DU145 cells. However, protein levels of Slug and Twist were not affected and only slightly decreased after osthole treatment. We also found that osthole attenuated the DNA-binding activity of Snail in the E-cad promoter region. Otherwise, we found that Snail was downregulated in osthole-treated PC3 xenografts and was positively correlated with the histological Gleason score, overall survival, and PSA level of prostate cancer patients. Snail is a zinc-finger transcription factor that can suppress *E-cad* gene expression via binding to E-box sequences in the proximal *E-cad* promoter [37]. Snail is aberrantly expressed in TGF-β- and tumor necrosis factor (TNF)-α-induced EMT, and functions as the key organizer [38]. Aberrant expression of Snail was reported to be associated with tumor recurrence and metastasis, and a poor prognosis in various human cancers [39, 40]. Taken together, our results support findings that Snail is a regulator of tumor metastasis in prostate cancer, and osthole regulates the expression of E-cad, at least partially, through transcriptional regulation by Snail. PI3K/Akt signaling and MAPKs are important downstream effectors in response to TGF-β-mediated EMT in cancers including AIPC [41, 42]. PI3K/Akt signaling was also reported to regulate Snail expression in AIPC cells [43]. Our present study indicated that osthole inhibits TGF-β-mediated Snail expression by targeting the Akt, ERK, and JNK pathways in AIPC cells. In addition, Rho proteins, cyclooxygenase (COX)-2, and Smad signaling are also important for TGF-β-mediated cell motility and the EMT [41, 42, 44], and the effects of osthole on these pathways in AIPC cells should be further elucidated in the future.

The newly identified small noncoding RNAs, miRNAs, belong to a novel class of gene regulators that control post-transcriptional regulation of genes by binding to complementary sequences in the 3′UTRs of target mRNAs. Deregulation of expression of miRNAs was reported in various human cancers to regulate the EMT. For example, the miR-200 family and miR-205 were shown to contribute to the EMT in cancer cells by directly targeting the transcriptional repressors of E-cad, ZEB1, and ZEB2 [45, 46]. MiR-9 is activated by MYC/MYCN-mediated E-cad downregulation resulting in metastases of neuroblastomas and breast tumors [47]. In addition to osthole-mediated transactivation inhibition of the *E-cad* gene, we further investigated which miRNAs might be involved in osthole-mediated *E-cad* gene expression and cell motility in AIPC cells. Combining data from our miRNA screening profiles and miRNA target databases, we found that osthole treatment of AIPC cells downregulated the expression of miR-23a-3p which revealed the suppressive effect on E-cad and promoting effect on cell motility.

MiR-23a-3p is highly expressed and acts as an oncogenic miRNA in various cancers [48]. MiR-23a/24/27a is an miRNA cluster located in chromosome 19p13.12 and can be regulated by MAPK signaling and TGF-β [48]. Previous reports indicated that miR-23a/24/27a functions as a growth-promoting and antiapoptotic factor in HCC cells [35], while miR-23a-3p was also shown to promote the growth of gastric adenocarcinoma cells and downregulate an interleukin-6 receptor [49]. Moreover, miR-23a-3p promotes the transition of colorectal cancer from the indolent to the invasive phenotype and the invasive ability of glioma cells by directly targeting HOXD10 [50]. We found that expression of miR-23a-3p was suppressed by osthole and induced by TGF-β in AIPC cells. We suggest that osthole may suppress TGF-β-mediated miR-23a-3p upregulation in AIPC cells. Furthermore, overexpression of miR-23a-3p decreased E-cad expression and significantly reversed osthole-mediated inhibition of cell invasion, while silencing of miR-23a-3p partially inhibited cell motility. Taken together, our report showed that miR-23a-3p regulates the TGF-β-induced EMT via E-cad suppression in AIPC cells, and osthole can significantly suppress this phenomenon. The role of miR-23a-3p in regulating E-cad in AIPC is consistent with that in a report on lung cancer [21].

**Figure 7:**
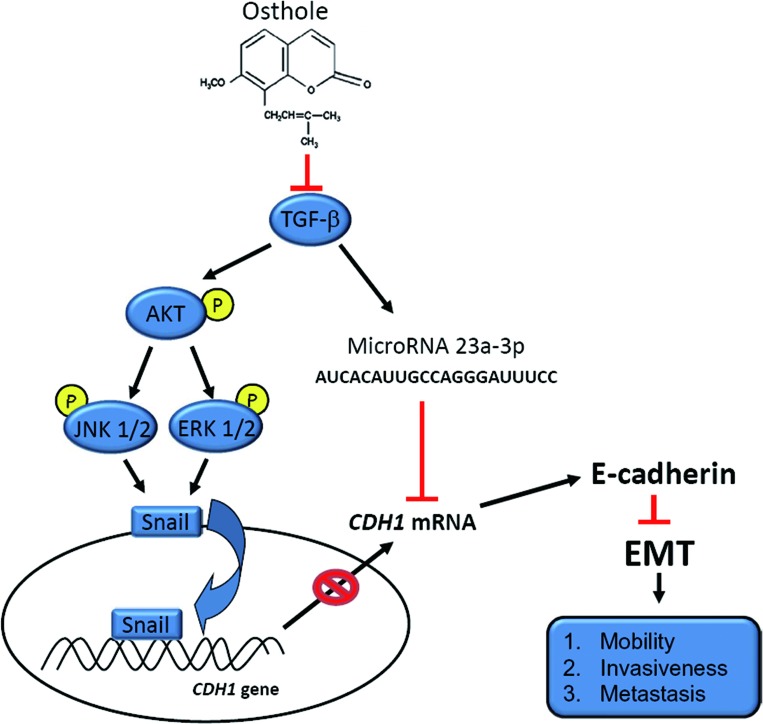
A working model showing the molecular signals underlying the ability of osthole to suppress the motility of androgen-independent prostate cancer (AIPC)

We suggest that osthole inhibits metastasis via transcriptional and epigenetic regulation of E-cad by respectively altering Akt/MAPK/Snail pathways and miR-23a-3p expression, which was initiated by inhibition of TGF-β production (Figure 7). These results may warrant clinical trials of osthole in AIPC.

## MATERIALS AND METHODS

### Materials

Osthole, PD98059, SP600125, and Ly294002 were purchased from Sigma-Aldrich (St. Louis, MO). Cell culture materials and fetal bovine serum (FBS) were obtained from Gibco-BRL (Gaithersburg, MD). An antibody specific for E-cad was obtained from Abcam (Cambridge, MA). Antibodies specific for TGF-β, β-catenin, Snail, Slug, and unphosphorylated or phosphorylated (p) forms of the corresponding Akt, c-Jun N-terminal kinase (JNK)1/2, extracellular signal-regulated kinase (ERK)1/2, and p38 were obtained from Cell Signaling Technology (Danvers, MA). Antibodies specific for vimentin and N-cad were purchased from BD Biosciences (San Jose, CA). Antibodies specific for Twist, matrix metalloproteinase (MMP)-9, MMP-2, Ki-67, α-tubulin, and β-actin were obtained from Santa Cruz Biotechnology (Santa Cruz, CA). The Mir-23a-3p mimic and inhibitor were purchased from GenePharma (Shanghai, China). The E-cad 3′UTR reporter was a gift from Dr. M. L. Kuo (National Taiwan University, Taipei, Taiwan). Unless otherwise specified, other chemicals used in this study were purchased from Sigma Chemical (St. Louis, MO).

### Cell culture

The PC3 and DU145 human AIPC cell lines were obtained from American Type Culture Collection (Manassas, VA). PC-3M, a highly metastatic subline derived from the hepatic metastasis of PC-3 [51], was provided by Dr. M. L. Kuo (National Taiwan University). PC3 and PC3-M cells were cultured in minimum essential medium (MEM) and DU145 cells were cultured in Dulbecco's modified Eagle medium (DMEM) supplemented with 10% FBS and a 1% antibiotic-antimycotic. Regular passaging was performed at 70%~80% confluence through trypsinization using 1× trypsin and 0.05% EDTA, followed by resuspension in complete medium. Cells were grown and maintained at 37°C in a 5% CO_2_ and 95% air atmosphere.

### Cell-viability assay

5×10^3^ Du145 or PC3 cells were plated in 96-well plates, treated with osthole (20~80 μM) for 24 h, and then subjected to a cell-viability assay (MTS assay; Promega, Madison WI) according to the manufacturer's instructions. Data were collected from three replicates.

### *In vitro* wound-closure assay

PC3 cells (3 × 10^5^ cells/well) or DU145 cells (4 × 10^5^ cells/well) were plated in 6-well plates for 24 h, wounded by scratching with a pipette tip, then incubated with α-MEM or DMEM medium containing 0.5% FBS, and treated with or without osthole (20~80 μM) for 24 h. Cells were photographed using a phase-contrast microscope (100×) as previously described [52].

### Transwell invasion assay

Migration and invasion assays were performed as described previously [53]. Briefly, a transwell invasion assay used 2 10^5^ cells plated in a Matrigel (BD Biosciences, Bedford, MA)-coated top chamber and incubated for 24 h (with a 24-well insert and a pore size of 8 μm; Corning Costar, Corning, NY). In this assay, cells which had been pretreated for 1 h with osthole (20~80 μM) were plated in medium without serum or growth factors, and medium supplemented with serum was used as a chemoattractant in the lower chamber. After 24 h of incubation, cells that had invaded through the pores were removed with a cotton swab. Cells on the lower surface of the membrane were fixed with methanol and stained with crystal violet. The number of cells invading through the membrane was counted under a light microscope (×40, three random fields per well).

### Flow cytometric analysis

DU145 cells were grown in DMEM supplemented with 10% FBS. After cells had grown to subconfluence, they were rendered quiescent and challenged with 10% FBS and vehicle or 60 μM osthole. After 24 h, they were harvested, washed twice with phosphate-buffered saline (PBS)/0.1% dextrose, and fixed in 70% ethanol at −20 °C. Nuclear DNA was stained with a reagent containing propidium iodide (PI; 50 mg/mL) and DNase-free RNase (2 U/mL) and measured using a fluorescence-activated cell sorter (FACS). The proportion of nuclei in each phase of the cell cycle was determined using WinMDI 2.9 DNA analysis software (The Scripps Research Institute, La Jolla, CA).

### Immunofluorescence microscopy

DU145 cells grown on cover slips were treated with or without osthole and fixed in 4% paraformaldehyde, permeabilized, and stained with a primary antibody against E-cad or N-cad, followed by incubation with a DyLight^TM^488-conjugated secondary antibody (Jackson Laboratories, West Chester, PA) to observe distributions of these proteins. Slides were examined and photographed using a Zeiss Axiophot fluorescence microscope (Carl Zeiss Microimaging, Gottingen, Germany). Nuclei were counterstained with 4′,6-diamino-2-phenylindole (DAPI).

### Chromatin immunoprecipitation (ChIP) analysis

A ChIP analysis was performed as described previously [54]. DNA immunoprecipitated with antibodies specific to Snail or the control, rabbit IgG, was purified and extracted using phenol-chloroform. Immunoprecipitated DNA was analyzed with a PCR or quantitative (q)PCR using specific primers which are described in supplementary data (Supplementary Table 1).

### Reverse-transcriptase polymerase chain reaction (RT-PCR)

Messenger (m)RNA was isolated and amplified as described previously [54]. Primer sequences are shown as supplementary data (Supplementary Table 1).

### Western blot analysis

Protein lysates were prepared as described previously [54]. A Western blot analysis was performed with primary antibodies for E-cad, β-catenin, N-cad, vimentin, Snail, Slug, Twist, MMP-2, MMP-9, TGF-β, Akt, p-Akt, ERK, p-ERK, p38, p-p38, JNK, p-JNK, α-tubulin, or β-actin.

### *In vivo* metastasis model

All animal work was performed in accordance with protocols approved by the Institutional Animal Care and Use Committee of Taipei Medical University. Age-matched male severe combined immunodeficient (SCID) mice, at 6~8 weeks old, were used in assays for tumor growth and metastasis in an orthotopic graft model. Luciferase-tagged PC3M cells (5 × 10^5^) were suspended in Matrigel, and injected into the prostate of SCID mice using a 30-gauge needle through a lower abdominal incision. Eight days after tumor cell injection, mice were randomized into experimental and control groups according to bioluminescence imaging from the Xenogen IVIS-200 system (Xenogen, Alameda, CA) such that treatment was initiated with similar mean tumor sizes in each group, and then mice were treated with different dosages (30 or 100 mg/kg) of osthole or the vehicle control every other day by intraperitoneal (IP) administration. The following day after osthole treatment, we used this live imaging device to mointor the tumor size and location. Twenty-one days after osthole treatment, animals were sacrificed, and tumor specimens were resected for immunohistochemical (IHC) staining.

### Patients and specimen preparation

Prostate cancer samples from patients were obtained with informed consent from Taipei Medical University-Wan Fang Hospital and Taipei Medical University Hospital in Taipei, Taiwan (Taipei Medical University-Wan Fang Hospital Institutional Review Board no. 99049). Pathology files of Wan Fang Hospital and Taipei Medical University Hospital were searched, and radical prostatectomy specimens with a pathologic diagnosis of prostatic adenocarcinoma were identified from March 1999 to December 2011. The pathologic diagnosis and Gleason scoring were microscopically reconfirmed by pathologists. Each case was pathologically staged using the 2002 American Joint Committee on Cancer TNM staging system. Paraffin-embedded, formalin-fixed surgical specimens from all patients were collected for IHC staining.

### Tumor IHC

All tumor specimens were embedded in paraffin blocks and cut in 4-μm sections. All specimens were deparaffinized and immersed in 10 mM sodium citrate buffer (pH 6.0) in a microwave oven twice for 5 min to enhance antigen retrieval. After washing, slides were incubated with 0.3% H_2_O_2_ in methanol to quench the endogenous peroxidase activity. Slides were washed with PBS and incubated with anti-Ki67, anti-Snail (monoclonal mouse anti-snail antibody, Biorbyt), and anti-mouse immunoglobulin G (IgG) antibodies for 2 h at room temperature. After washing in PBS, slides were developed with a VECTASTAIN ABC (avidin-biotin complex) peroxidase kit (Vector Laboratories, Burlingame, CA) and a 3,3,9-diaminobenzidine (DAB) peroxidase substrate kit (Vector Laboratories) according to the manufacturer's instructions. All specimens were deparaffinized and stained with hematoxylin and eosin (H&E) which was used as a light counterstain. IHC results of Ki67 were scored by taking into account the percentage of positive detection. IHC results of Snail were classified into two groups according to the intensity and extent of staining: the intensity was scored semiquantitatively as 0, negative; 1 point, weakly positive; 2 points, moderately positive; or 3 points, strongly positive. To determine the extent of Snail expression, 1000 consecutive malignant cells were counted in the area of strongest staining. The extent of Snail staining was semiquantitatively scored as 0, positive in < 1% of cells; 1 point, positive in 1%~25% of cells; 2 points, positive in 25%~50% of cells; 3 points, positive in 50%~75% of cells; or 4 points, positive in 75%~100% of cells. We then developed a Snail image score by multiplying the intensity score (0~3 points) by the extent score (0~4 points) to represent the expression of Snail in cancer tissues. Low and high expression levels of Snail were respectively defined as 0~6 and 8~12 points.

### DNA transfection

The myr-Akt plasmid was provided by Dr. C. C. Chen (National Taiwan University). To overexpress Akt, semiconfluent cultures of DU145 cells in a 6-mm^2^ Petri dish were transfected with 5 μg of an empty or expression vector (pcDNA3.1) using Invitrogen Lipofectamine 2000 Transfection Reagent. After incubation for 24 h, cells were analyzed for the expression of exogenous Akt by immunoblotting using an anti-Akt antibody.

### 3′UTR luciferase reporter assay

DU145 cells were seeded at a concentration of 5 × 10^4^ cells per well in 6-well cell culture plates. After 24 h of incubation, cells were co-transfected with an miR-23a-3p mimic or negative control and vector containing the E-cad 3′UTR. The pRL-TK Renilla control vector (0.5 μg) (Promega) was also co-transfected as an internal control for transfection efficiency. GenMuteTM siRNA & DNA Transfection Reagent (SignaGen Laboratories, Ijamsville, MD) was used for this transfection process according to the manufacturer's instructions. Cells were harvested 48 h after transfection and analyzed for luciferase activity using the Dual-Luciferase Reporter Assay System (Promega).

### TaqMan miRNA real-time RT-PCR

To determine expressions of miR-146a, 22-3p, and 23a-3p from AIPC cell lines, we used a TaqMan MicroRNA Assay kit (Applied Biosystems, Carlsbad, CA) following the manufacturer's protocol. Briefly, 10 ng of RNA from each cell line was reverse-transcribed using 10 μl of an RT mixture containing dNTPs, RT, an RNase inhibitor, and 3 μl of the respective primer. The mixture was incubated at 16 °C for 30 min, 42 °C for 30 min, and 85 °C for 5 min. Real-time PCRs were then carried out in a total volume of 20 μl of reaction mixture containing 2 μl of the RT product, 10 μl of 2 × TaqMan universal PCR master mix, 7 μl of water, and 1 μl of the TaqMan assay probe. All reactions, including the controls, were performed in triplicate. Relative expressions of miRNAs were analyzed using the Ct method and were normalized to RNU6B expression.

### Statistical analysis

Values are presented as the mean ± standard error (SE). All statistical analyses were performed using the Statistical Package for Social Science software, vers. 16 (SPSS, Chicago, IL). Data were analyzed using a Student's *t*-test when two groups were compared. A one-way analysis of variance (ANOVA) followed by Tukey's post-hoc test was used to analyze three or more groups. Statistical analyses of clinicopathological data were performed by a Chi-squared and Fisher's exact tests. The Kaplan-Meier method was used to compare the survival time among groups. *p* values of < 0.05 were considered statistically significant.

## SUPPLEMENTARY MATERIAL FIGURES AND TABLE


