# Supplementary material for: By inhibiting snail signaling and miR-23a-3p, osthole suppresses the EMT-mediated metastatic ability in prostate cancer
Source: Oncotarget. 2015 May 22;6(25):21120–36. doi: 10.18632/oncotarget.4229 (PMC4673254; doi:10.18632/oncotarget.4229)
Supplement: Supplementary file 1 [file oncotarget-06-21120-s001.pdf]

By inhibiting snail signaling and miR-23a-3p, osthole suppresses the EMT-mediated metastatic ability in prostate cancer

Supplementary Material

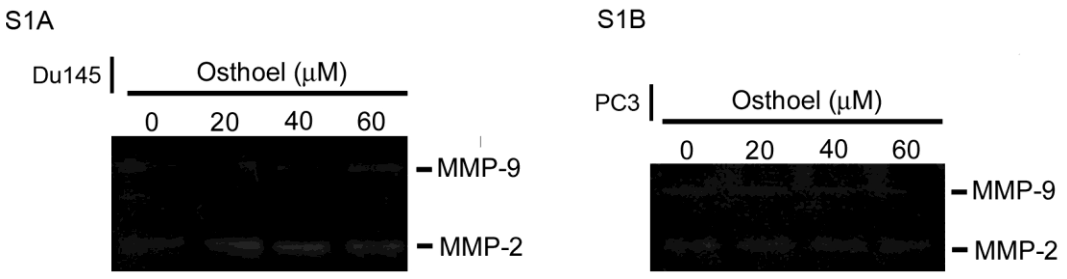

Figure S1: Effects of osthole on the activities of MMP-2 and MMP-9. DU145 cells (A) or PC3 cells (B) were treated with different concentrations of osthole for 24 h and then subjected to zymography to analyze the activities of MMP-2/9.

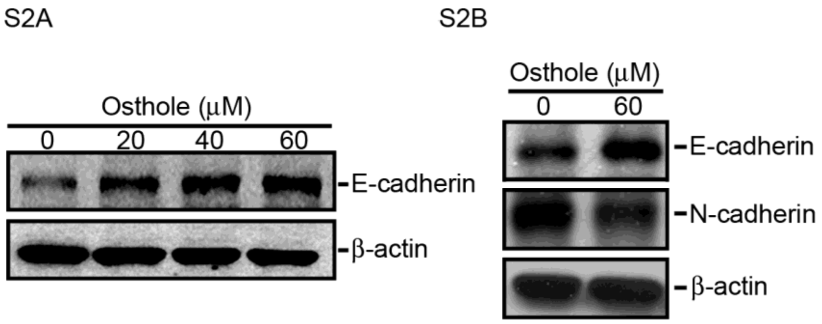

Figure S2: Effects of osthole on the expressions of EMT-related markers. PC3 cells were treated with different concentrations of osthole (0~60 μM) (A) or 60 μM osthole (B) for 24 h and then subjected to a Western blot analysis to analyze the expression of EMT-related markers, E-cadherin and N-cadherin.

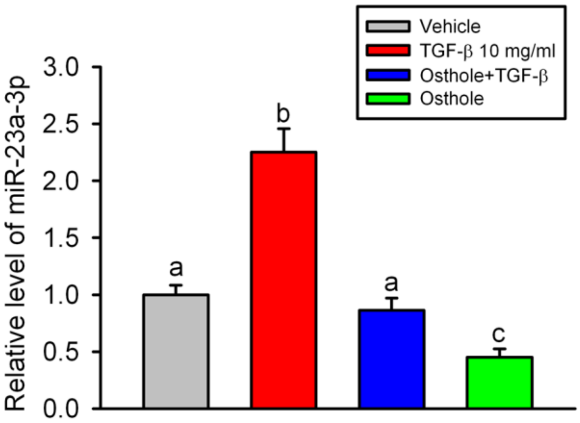

Figure S3: Effect of osthole on the TGF-β-induced expression of mir-23a-3p. Serum-starved DU145 cells were pretreated with osthole (60 μM) for 1 h followed by a 6-h TGF-β (10 ng/ml) incubation and then subjected to real-time PCR to analyze the expression of mir-23a-3p. Data were analyzed using a one-way ANOVA with Tukey's post-hoc tests at 95% confidence intervals; different letters represent different levels of significance.

Table S1: Primers list for RT-PCR and qPCR assay.

Primers used for RT- PCR

Gene	Species	Sequence (5' to 3')
Snail	Human	5'-TTTACCTTCCAGCAGCCCTA-3' (forward)
		5'-GAGATCCTTGGCCTCAGAGA-3' (reverse)
GAPDH	Human	5'-CGGAGTCAACGGATTTGGTCGTAT-3' (forward)
		5'-AGCCTTCTCCATGGTTGGTGAAGAC-3' (reverse)

Primers used for ChIP

Gene	Species	Sequence (5' to 3')
E-cad promoter	Human	5'- TAGAGGGTCACCGCGTCTAT-3' (forward)
Snail binding site		5'- TCACAGGTGCTTTGCAGTTC-3' (reverse)

Gene	Species	Sequence (5' to 3')
MiR-23a-3p	Human	5'- AUCACAUUGCCAGGGAUUUCC-3'
MiR-146a	Human	5'-UGAGAACUGAAUUCCAUGGGUU-3'
Mir-22-3p	Human	5'-AAGCUGCCAGUUGAAGAACUGU-3'
RNU6B	Human	5'-CGCAAGGATGACACGCAAATTCGTGAAGCGTTCCATATTTTT-3'
